# Strangulated Hernia Reduced during Vomiting

**Published:** 1852-03

**Authors:** 


*Strangulated Hernia Reduced during Vomiting.—L'Union Médicale* mentions that Dr. Kuttlinger, of Erlangen, in Germany, tried the taxis upon a woman sixty-four years of age, whose crural hernia was strangulated; but without success. The patient was soon attacked with vomiting, and whilst she was making efforts, Dr. Kuttlinger seized the tumor, pressed it with some force, and succeeded in reducing it in the very midst of the straining. Three months afterwards strangulation occurred again, the taxis was tried in vain, and reduction was effected exactly in the same manner as before.—*Ibid.*

